# Sensory information and the perception of verticality in post-stroke patients. Another point of view in sensory reweighting strategies

**DOI:** 10.1371/journal.pone.0199098

**Published:** 2018-06-29

**Authors:** Wim Saeys, Nolan Herssens, Stijn Verwulgen, Steven Truijen

**Affiliations:** 1 University of Antwerp, Department of Rehabilitation Sciences and Physiotherapy, Wilrijk, Belgium; 2 Rehabilitation Hospital Revarte, Wilrijk, Belgium; 3 University of Antwerp, Department of Product Development, Antwerp, Belgium; Ludwig-Maximilians-Universitat Munchen, GERMANY

## Abstract

**Introduction:**

Perception of verticality is highly related to balance control in human. Head-on-body tilt <60° results in the E-effect, meaning that a tilt of the perceived vertical is observed contralateral to the head tilt in the frontal plane. Furthermore, somatosensory loss also impacts the accuracy of verticality perception. However, when several input sources are absent or biased, less options for sensory weighting and balance control occur. Therefore, this study aims to identify the E-effect and assess the effect of somatosensory loss on the extent of the E-effect.

**Methods:**

All patients with a first stroke admitted to a Belgian rehabilitation hospital were eligible for inclusion. Patients aged above 80 with other neurological and orthopaedic impairments as well as brainstem, cerebellar or multiple lesions were excluded. In addition, patients with visuospatial neglect and pusher behaviour were also excluded as this can affect verticality perception. The Rivermead Assessment of Somatosensory Performance (RASP), the Subjective Visual (SVV) and Subjective Postural (SPV) Vertical Test were administered.

**Results:**

In total, 37 patients were included in the analysis of which 24 patients completed both SVV and SPV assessment. Results show that the E-effect occurred in our sample of stroke survivors for both SVV and SPV. In addition, the presence of somatosensory loss will increase the E-effect in both SVV as SPV assessment. A significant difference in verticality perception was noted for both SVV and SPV between the group with no (SVV: 5.13°(6.92); SPV: 0.30°(1.85)) and highly severe (SVV: 10.54°(13.19); SPV: 5.96°(9.27)) sensory loss.

**Conclusions:**

The E-effect occurs in stroke subjects and increases when patients experience somatosensory loss. This suggests that the lack of available afferent information impede estimation of verticality. Therefore, stroke survivors have fewer alternative input sources as a result of impairments, leading to fewer options about sensory reweighting strategies and balance recovery after perturbations.

## Introduction

Postural control emerges from the interaction between the task, the environment and the individual. Within the individual, an efficient interaction between motor, sensory and neural systems is needed in order to maintain postural control.[1] One of the neural processes is the integration of afferent information such as visual, vestibular (otolith organs and semicircular canals) and somatosensory (muscle and joint proprioceptors, skin pressure sensors, truncal somatosensors in the kidneys, mechanoreceptors in large vessels) input to enhance self-orientation in the gravitational space.[2] Humans should be able to align their body vertically with the gravitational vector to ensure axial extension of the body, keeping the centre of pressure within the base of support. To orientate the whole body in space, different reference frames are constructed based on several input sources from head and trunk. Dissociating head- and trunk-based reference frames and their specific sensory input sources to estimate the direction of gravity, underlines the eminent role of the vestibular organs in sensing gravity.[3, 4] However, this does not suggest that internal verticality is solely based on sensory systems in the head. Clearly, verticality perception remains the result of a multisensory integration of various sensory input signals within parietotemporal cortical areas [5–7] and relevant contributions by somatosensory sensors in the neck and trunk (e.g., truncal somatosensors, skin pressure sensors, muscle and joint proprioceptors, and kidney graviceptors) have been reported.[8–13]

Both vestibular and somatosensory deficits may result in changes in verticality perception. These changes in verticality estimate accuracy are likely related to how the brain weights different sensory signals. Various studies have shown that humans integrate different sensory information in a way consistent with a weighted linear combination of perceptual estimates from the individual sensory input sources. [14–16] This means that the weighting of each signal is proportional to the signal’s relative reliability, such that a less-reliable signal is given less weight in verticality estimates e.g. vision in complete darkness. This reweighting of sensory information is crucial for the generation of appropriate postural responses in different tasks and environments. However, sensory reweighting is not always adequate when certain input sources are altered. In verticality perception, when the roll tilt of the head is less than 60°-70°, a contralateral deviation of the subjective vertical has been reported.[17] This is called the E-effect and has been observed in both the Subjective Visual Vertical test (SVV) and the Subjective Postural Vertical test (SPV).[18, 19] Furthermore, studies have shown that also somatosensory loss has a negative impact on perceiving verticality in patients after stroke.[20]

In the case of a deteriorated signal-to-noise ratio of vestibular signals (e.g., due to a tilted head-on-body position or uni- or bilateral vestibular loss), visual and somatosensory input is expected to be weighted more strongly by the brain to compute head-on-body position and body orientation in space [2, 14]. However, when somatosensory input is impaired, this will further lead to a hindered estimation of the earth vertical by the absence of alternative input sources.[21] As a result, this will reflect in an increase of the E-effect which is of major importance since several studies have shown that verticality perception is highly related to balance [22, 23]. In this study, we will explore verticality perception and sensory reweighting strategies in stroke subjects. At first, the presence of the E-effect will be investigated in our sample of stroke subjects. Secondly, we will investigate the effect of somatosensory loss on the extent of the E-effect. Therefore, we will assess stroke patients with a lesion in parietotemporal regions and regions supplied by the middle cerebral artery, to investigate the sensory weighting and multisensory integration of various sensory input signals.

## Patients and methods

### Study design

A cohort study was designed to investigate whether the E-effect occurs in people after stroke. In addition, the effect of somatosensory loss on the extent of the E-effect will be investigated to provide further insights in the sensory reweighting strategies. Ethical approval was given by the ethical committee with registration number B300201630358, University Hospital Antwerp, Belgium, in accordance with the Declaration of Helsinki 1975, revised Hong Kong 1989.

### Patients

Consecutive patients were recruited from the stroke population of the rehabilitation hospital Revarte, Antwerp, Belgium. All patients with a history of first stroke attending a rehabilitation program were eligible for inclusion. Patients who had an age above 80; other neurological and orthopaedic impairments as well as brainstem, cerebellar or multiple lesions were excluded. Only strokes with an ischemic or hemorrhagic etiology were included. Patients were also excluded when the subjects had pre-existing co-morbid conditions that might have affected vision and somatosensory function. Also sufficient range of motion (ROM) of the cervical spine must be present, which was assessed during inclusion. A ROM of the head in relation to an upright sternum of minimum 35° must be obtained measured with a goniometer. In addition, patients with visuospatial neglect and pusher behaviour were also excluded as this can affect verticality perception.[24–27] Pusher behaviour was examined by a neuropsychologist and the use of the Scale of Contraversive Pushing (SCP)[28]. In addition, patients had to perform the assessment within three months post-stroke. Prior to inclusion, the participants were asked whether they understood the instructions of tests and to sign a written informed consent.

## Outcome measures

### Rivermead assessment for somatosensory performance

The Rivermead Assessment for Somatosensory Performance (RASP) measures different somatosensory modalities on the face, hands and feet and has been noted to be a reliable and standardized assessment for somatosensory performance. Six tests are administered on each of the ten (five left and five right) test regions on the face (cheeks); palmar and dorsal of the hands and plantar and dorsal of the feet, two tests are administered only on the face and palm of the hands. During testing, eyes of the participants are closed. These eight tests can be divided into six primary and two secondary tests of sensation. The primary tests measure sharp/dull discrimination, surface pressure touch, surface localization, temperature discrimination, proprioception movement discrimination and proprioception direction discrimination. Each primary test is scored on a total of 60 points with a total of 360 points for all primary tests. Six trials are executed on each of the ten test regions, for two of the trials, sham trials were given. Sham trials increase the patient’s internal reliability. Patients were excluded from the statistical analysis if they had more than five false positive replies, suggested by Winward et al.[29]

## Subjective Visual Vertical (SVV)

The Difra Vertitest type D107201 (Difra, Welkenraedt, Belgium) was used for SVV assessment. The device has an accuracy of 0.1°. A laser bar was projected vertically at a distance of 2.5m on an opposing wall with the center of rotation of the laser bar on an altitude of 1.5m. The device was calibrated in this position, approximating the average altitude of the participants’ eyeline when seated. The patients are seated in front of the device on a fixed chair without any arm- or backrests. The room was darkened and five minutes of waiting period was given allowing the subject to adjust the darkness. Both researcher and participant obtained a remote control to allow rotating the laser bar either clockwise (right) or counter clockwise (left). The researcher’s remote control showed a display with the amount of deviation in relation to the earth’s gravitational vector. The researcher made the laser bar invisible and rotated it in a specific angle in relation to the earth vertical. Subsequently, the line was shown after which the patient had to place the line in upright position again with his nonhemiplegic hand on the remote control. The amount of deviation of each starting roll position was different for each trial. A specified order was followed: first the line was placed in 20° counter clockwise, 10° clockwise, 5° counter clockwise and 0° according to the earth vertical, followed by 5° clockwise, 10° counter clockwise and finally 20° clockwise. This series was executed three times. During the first series the patient was asked to hold the head in normal upright position, followed by a series with the head actively tilted to the left as far as possible (while the head was tilted the subjects needed to keep their trunk upright) and finally a series with the head actively tilted to the right side as far as possible. The clockwise rotation is represented by a positive number and the counter clockwise rotation as a negative number. Head-on-body tilt was tactilely controlled by the researcher to decrease variability in head-on-body tilts and neck proprioceptive information. All patients had a ROM of the head in relation to the sternum between 35 and 45°.

## Subjective Postural Vertical (SPV)

The rotation chair works on hydraulic pumps and has a height of 1m. On the back of the chair, a Mitutoyo digital protractor pro 3600 (Belgium) was mounted. This allowed measurement of the deviation in relation to the earth vertical with an accuracy of 0.01°. Both the researcher and patient were given a remote to rotate the chair clockwise (right) and counter clockwise (left). Movements were restricted in the frontal plane. Before the assessment started the patient was blindfolded, depriving the subjects of visual information when readjusting the chair to earth vertical. The researcher rotated the chair as in the procedure of SVV (starting roll position of the chair). The head-on-body position is similar as in the SVV procedure. The subject had to place the chair in upright position again by placing the seating surface of the chair horizontal. The patient used his non-hemiplegic hand on the remote control. The clockwise rotation is shown positively and the counter clockwise rotation negatively.

## Statistical analysis

Statistical Package for Social Sciences, SPSS 22 (SPSS inc, Chicago, IL) for windows was used for statistical analysis.

Descriptive statistics were used for age, days post stroke, lesion side and RASP scores. Normality was checked by the Kolmogorov-Smirnov test.

To allow comparison of the E-effect on both sides, corrected SVV and SPV scores (denoted SVVc and SPVc) are calculated by multiplying the raw SVV and SPV scores of patients with right head-on-body tilt with -1. Since right-head-on body positions have a counter clockwise deviation (negatively) and left head-on-body positions clockwise (positively), the average of both E-effects would balance each-other out.

In order to assess sensory loss based on the RASP, only the total scores of the primary tests were used for the analysis as proposed by Winward et al.[29] Each subtest had a maximum score of 60 points, setting the total score on 360 points. Because the studied stroke population had only unilateral lesions and hemiparesis and the RASP assesses bilaterally sensory loss, subjects were post hoc divided into five groups (ordinal scores) starting from a total score of 180 points. An ordinal score of 4 meant highly severe sensory loss (total score of 180–216), a score of 3 severe sensory loss (total score 217–252), a score of 2 moderate sensory loss (total score of 253–288), a score of 1 mild sensory loss (total score of 289–324) and a score of 0 no sensory loss (total score of 325–360).[20]

To compare the mean SVV, SVVc, SPV and SPVc scores for each tilted (clockwise–counter clockwise in the frontal plane) head-on-body position, an independent sample t test was used.

The relationship between somatosensory loss, head-on-body tilt and perception of verticality was analysed using the ordinal scores based on the total RASP score, head-on-body tilt and SVV, SVVc, SPV and SPVc. This analysis was performed by a univariate ANOVA as data were normally distributed.

Another way to analyse the relationship between somatosensory loss, head-on-body tilt and perception of verticality was a one-way ANOVA test to analyse the relationship between the mean SVVc, SPVc and primary RASP scores.

Post hoc analysis was performed using the Bonferroni test for normally distributed data.

All data are fully available without restriction at Dryad Digital Repository. The DOI is doi:10.5061/dryad.9b031.

## Synthesis of results

Thirty-seven patients (22 men and 15 women), with a mean age of 62.43 (± 13.26) years, were included. Time from stroke onset ranged from 8 till 85 days with a mean of 38.05 (± 21.17) days post-stroke. Fifteen patients had a left-hemisphere lesion and twenty-two patients a right-hemisphere lesion. There were no patients with bilateral lesions. Descriptives of each individual, all patients combined and of each group based on sensory loss severity are shown in Tables 1 and 2. Out of the thirty-seven patients, three patients were unable to complete the SVV test-protocol and ten patients were unable to complete the SPV test-protocol due to safety issues or fear of falling. Twenty-four subjects completed both protocols (SVV and SPV).

**Table 1:** Clinical and demographic data.

Patient	Age in years	Sex	Days since lesion	Lesion side	I/H	Lesion localisation	SCP	RASP total primary scores	Sensory loss group
1	46	M	18	R	I	Acm	1.75	269	3
2	60	M	16	R	I	Acm	0	309	2
3	57	M	22	R	I	Acm	0	341	0
4	58	M	23	R	I	Acm	1.25	253	3
5	79	F	76	R	I	Acm	0	224	4
6	55	F	58	R	I	Ic	0	269	3
7	74	M	31	L	H	Acm	0	354	0
8	61	F	13	L	I	Ic, Th	0	360	0
9	53	M	16	L	I	Acm	0	334	1
10	69	F	40	R	I	Pa	1	211	3
11	50	F	25	R	I	Nl, Ic	0	359	0
12	63	M	15	R	I	Nl	0	346	0
13	71	F	8	R	I	Acm	0	224	4
14	78	M	57	R	I	Acm	0	246	3
15	68	F	46	L	I	Ic	0	244	3
16	69	F	28	L	I	Acm	0	341	0
17	69	M	21	R	I	Acm	1.5	211	4
18	58	F	52	R	H	Fr, Pa	1	310	2
19	48	F	58	R	I	Acm	1.75	207	4
20	35	M	44	R	H	Pa, Te	0	239	3
21	66	M	27	L	I	Acm	0	328	0
22	39	M	63	L	I	Acm	0	358	0
23	53	F	20	L	I	Fr, Pa, Te	0	327	0
24	68	M	26	R	I	Nc	1.75	336	0
25	79	F	45	L	I	Fr, Pa	0	351	0
26	76	M	37	R	I	Bg	0	331	0
27	51	F	40	L	H	Pa	0	298	2
28	75	M	29	L	I	Acm	0	322	1
29	77	F	19	L	I	Nl	0	352	0
30	79	M	42	L	I	Acm	0	347	0
31	34	M	57	L	I	Acm	0	316	1
32	73	M	81	R	I	Acm	1.5	202	4
33	58	M	85	R	I	Th, Pa	0	356	0
34	44	M	83	L	I	Nl	0	215	4
35	80	F	41	R	I	Pa, Te	0	334	0
36	57	M	21	R	I	Th, Pa	0	344	1
37	80	M	25	R	H	Acm	0	321	0

M: Male; F: Female; R: Right; L: Left; I: Ischemic; H: Hemorrhagic; ACM: Medial cerebral artery; Ic: Internal capsule; Nl: Nucleus lentiformis; Th: Thalamus; Nc: Nucleus caudatus; Bg: Basal ganglia; Fr: Frontal cortex; Pa: Parietal cortex, Te: Temporal cortex; SCP: Scale of Contraversive Pushing; RASP: Rivermead Assessment of Somatosensory

**Table 2:** Descriptives of all patients and in separate groups based on sensory loss.

Outcome measure	All Patients (N = 37) Mean (SD)	Group 0 (N = 17) Mean (SD)	Group 1 (N = 4) Mean (SD)	Group 2 (N = 3) Mean (SD)	Group 3 (N = 7) Mean (SD)	Group 4 (N = 6) Mean (SD)
Age (years)	62.43 (13.26)	66.47 (12.21)	54.75 (16.82)	56.33 (4.73)	58.43 (14.73)	64.00 (14.39)
Sex (m/f)	22/15	10/7	4/0	1/2	4/3	3/3
Post-stroke (days)	38 (21.17)	33.18 (18.30)	30.75 (18.30)	36.00 (18.33)	40.86 (15.45)	54.50 (32.48)

Group 0: No sensory loss; Group 1: Mild sensory loss; Group 2: Moderate sensory loss; Group 3: Severe sensory loss; Group 4: Highly severe sensory loss.

### Subjective Visual Vertical (SVV)

The head-on-body positions influenced the results of the SVV scores (*F* = 222.15; *df* = 1; p < 0.001; t-test). The SVV deviated to the opposite side of the starting head-on-body position. When the subjects were asked to tilt the head to the left while keeping the trunk upright, the SVV deviated clockwise (+7.23° ± 9.96°). When they were asked to tilt the head to the right while keeping the trunk upright, the SVV deviated counter clockwise (-6.99° ± 8.78°).

The severity of sensory loss also influenced the test results when considering the corrected SVV scores (*F* = 8.30; *df* = 4; p < 0.001, t-test). When sensory loss is more severe, the deviation of the SVV in relation to the earth vertical will increase. (Table 3)

**Table 3:** Total corrected Subjective Visual Vertical (SVV) scores and different head-on-body tilt (corrected) SVV scores of all patients and in separate groups based on sensory loss.

Outcome measure	All Patients (N = 34) Mean (SD)	Group 0 (N = 17) Mean (SD)	Group 1 (N = 4) Mean (SD)	Group 2 (N = 3) Mean (SD)	Group 3 (N = 6) Mean (SD)	Group 4 (N = 4) Mean (SD)
SVVc Total (degrees)	6.58 (8.96)	5.13 (6.92)	5.35 (3.70)	4.73 (4.26)	9.83 (12.61)	10.54 (13.19)
SVV Left (degrees)	7.23 (9.96)	5.78 (8.45)	8.10 (5.95)	3.62 (4.28)	8.96 (14.00)	12.61 (12.54)
SVVc Right (degrees)	6.99 (8.78)	5.89 (7.58)	5.85 (3.89)	5.84 (4.02)	10.69 (11.16)	8.11 (13.36)

Group 0: No sensory loss; Group 1: Mild sensory loss; Group 2: Moderate sensory loss; Group 3: Severe sensory loss; Group 4: Highly severe sensory loss. SVV Left: Subjective Visual Vertical with left head-on-body tilt; SVVc Right: Corrected Subjective Visual Vertical with Right head-on-body tilt.

**Fig 1:**
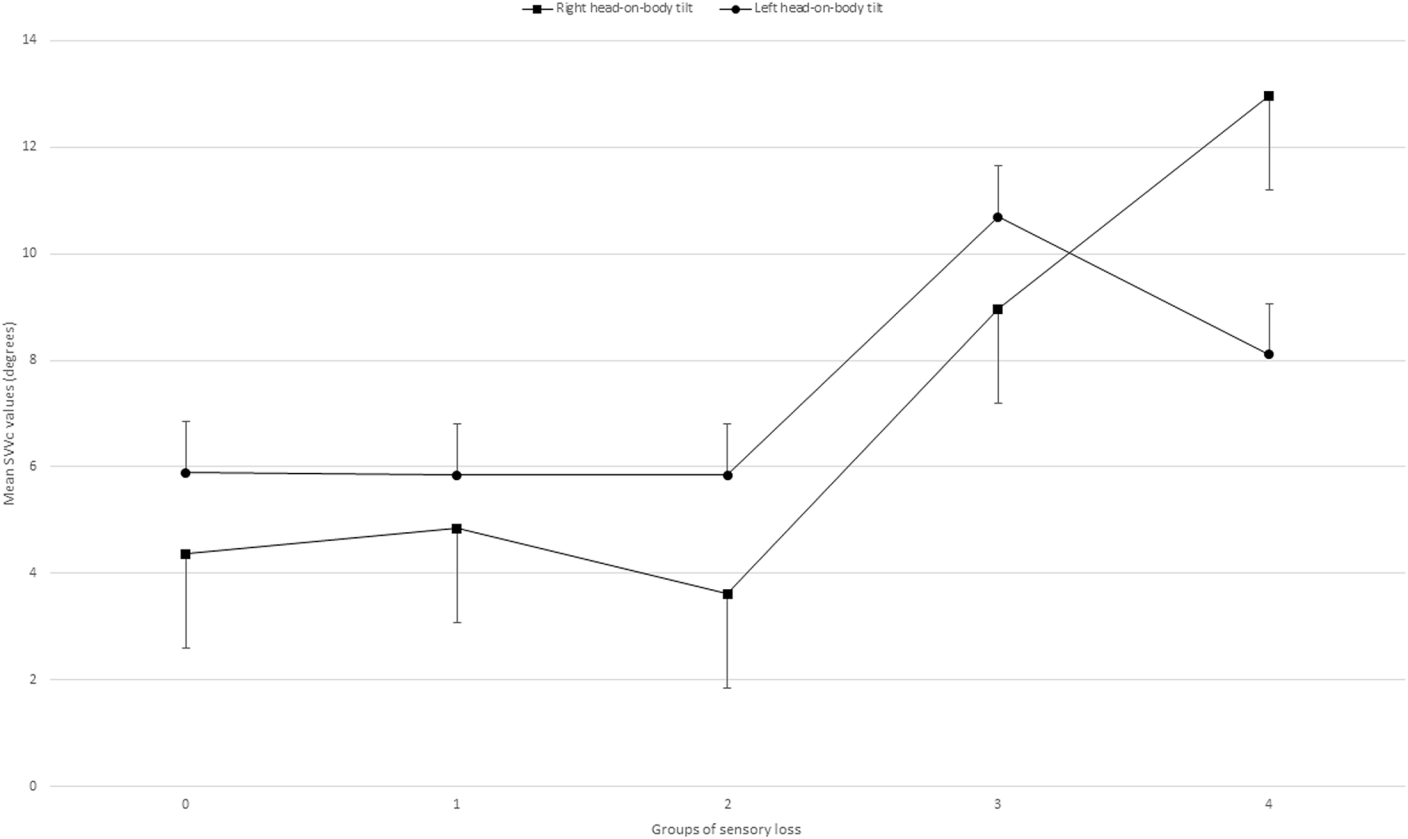
Corrected SVV scores of the groups of sensory loss. Group 0: No sensory loss; Group 1: Mild sensory loss; Group 2: Moderate sensory loss; Group 3: Severe sensory loss; Group 4: Highly severe sensory loss. SVVc: Corrected right head-on-body tilt scores of the Subjective Visual Vertical.

As previously mentioned, the SVV deviates to the opposite side to the head-on-body position. When the severity of sensory loss is analysed, using the 5-point RASP scores, an increase in SVV deviation was found when the sensory loss is more severe. A combined effect of head-on-body tilt and severity of sensory loss is shown (*F* = 5.415; *df* = 4; p < 0.001, uni-anova) indicating that the E-effect increases when more sensory loss is present (Fig 1).

Post hoc analysis of the 5-point RASP scores considering the SVVc results indicated that group 0 differed significantly from group 3 (p < 0.001, Bonferroni) and group 4 (p < 0.001, Bonferroni). Group 1 differed significantly from group 3 (p = 0.029, Bonferroni) and group 4 (p = 0.016, Bonferroni). Group 2 significantly differed from group 3 (p = 0.020, Bonferroni) and group 4 (p = 0.011, Bonferroni).

### Subjective Postural Vertical (SPV)

Analysis showed an effect of head-on-body positions on the SPV scores (*F* = 69.01; *df* = 1; p < 0.001; t-test). Similar as for the SVV, the SPV in general (SPVc total) deviated to the opposite side of the starting head-on-body position. As tilting the head to the left or right with the trunk being held in an upright position resulted in mean SPV scores of 1.65° ± 4.61° and -1.67° ± 5.65°, respectively.

As for the SVV, the severity of sensory loss only influenced the test scores when considering the corrected SPV scores (*F* = 16.29; *df* = 4; p < 0.001; t-test). The deviation of the SPV will increase when the sensory loss is more severe. (Table 4)

**Table 4:** Total corrected Subjective Postural Vertical (SPV) scores and different head-on-body tilt (corrected) SPV scores of all patients and in separate groups based on sensory loss.

Outcome measure	All Patients (N = 27) Mean (SD)	Group 0 (N = 12) Mean (SD)	Group 1 (N = 4) Mean (SD)	Group 2 (N = 3) Mean (SD)	Group 3 (N = 5) Mean (SD)	Group 4 (N = 3) Mean (SD)
SPVc total (degrees)	1.66 (5.14)	0.30 (1.85)	0.22 (2.61)	1.47 (3.16)	3.68 (7.33)	5.96 (9.27)
SPV Left (degrees)	1.65 (4.61)	0.47 (1.78)	0.54 (2.81)	1.50 (3.07)	3.73 (5.69)	4.62 (9.43)
SPVc Right (degrees)	1.67 (5.65)	0.11 (1.91)	-0.10 (2.39)	1.44 (3.32)	3.63 (8.75)	7.38 (9.12)

Group 0: No sensory loss; Group 1: Mild sensory loss; Group 2: Moderate sensory loss; Group 3: Severe sensory loss; Group 4: Highly severe sensory loss. SPV Left, Subjective Postural Vertical with Left head-on-body tilt; SPVc Right, Corrected Subjective Postural Vertical with Right head-on-body tilt.

**Fig 2:**
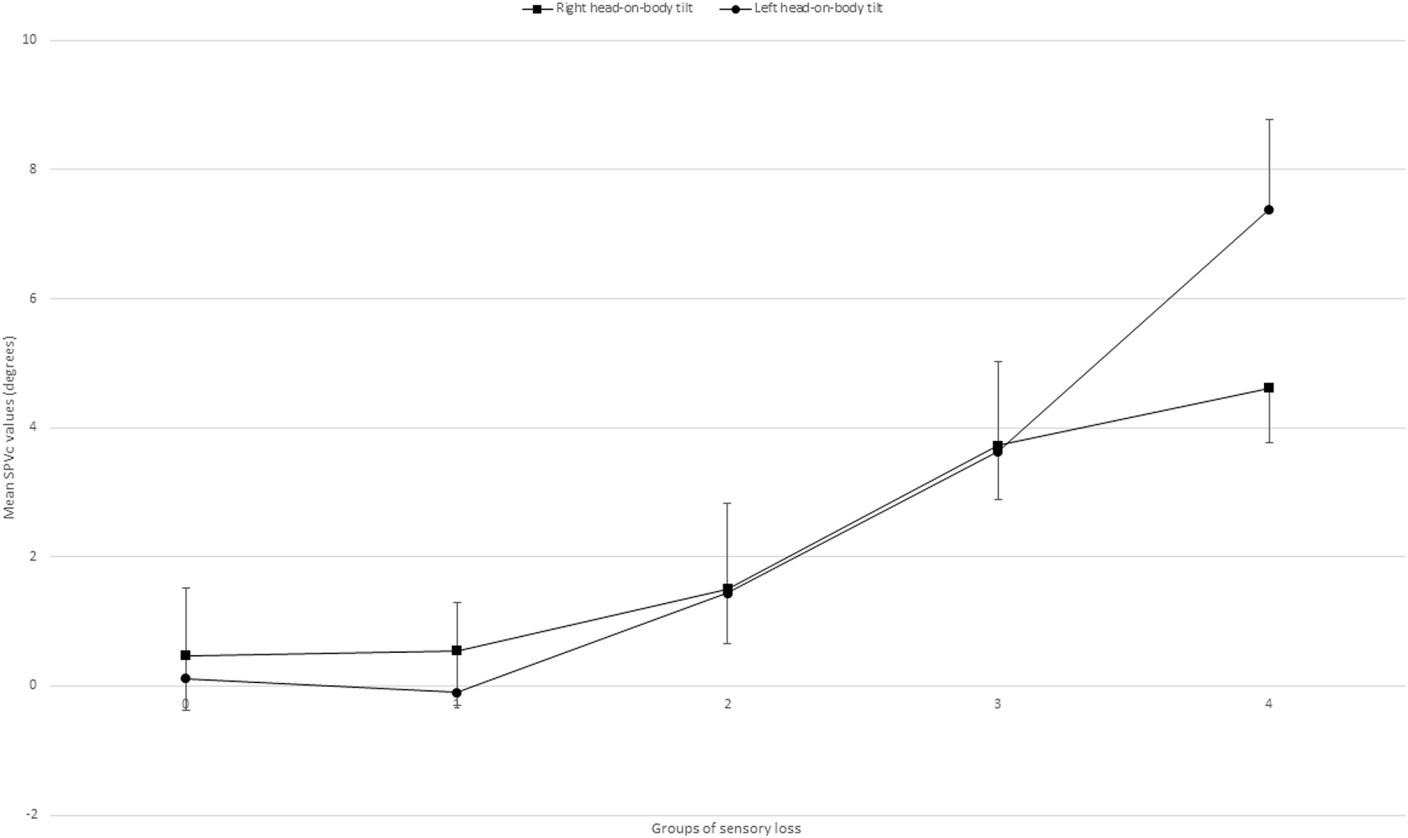
Corrected SPV scores of the groups of sensory loss. Group 0: No sensory loss; Group 1: Mild sensory loss; Group 2: Moderate sensory loss; Group 3: Severe sensory loss; Group 4: Highly severe sensory loss. SPVc: Corrected right head-on-body tilt scores of the Subjective Postural Vertical.

When starting head-on-body tilt and severity of sensory loss were combined, a significant effect was seen with *F* = 16.29; *df* = 4; p < 0.001 (uni-anova) indicating that the E-effect increases when more sensory loss is present (Fig 2).

Post hoc analysis of the 5-point RASP scores considering the SVVc results indicated that Group 0 differed significantly from group 3 (p < 0.001, Bonferroni) and group 4 (p < 0.001, Bonferroni). Group 1 differed significantly from group 3 (p = 0.001, Bonferroni) and group 4 (p < 0.001, Bonferroni). Group 2 differed significantly from group 4 (p < 0.001, Bonferroni).

## Discussion

In this study we explored verticality perception and sensory reweighting strategies in stroke subjects. At first, the E-effect occurred in our stroke subjects in general, which means that a contralateral deviation of SVV and SPV is seen when the head is tilted in the roll plane. Secondly, an effect of somatosensory loss on the extent of the E-effect can be observed. This means that a lower RASP score in combination with a tilted head-on-body position is related to a larger deviation of perceived verticality opposite to the head-on-body tilt in both the SVV and SPV. In the SPV measurements, group 0 and 1 can realign their body very accurately with a mean around the midline.

However, in our study large standard deviations (SD) have been observed. In literature, SD’s computed from repetitive trials of verticalization tests with the head in the same position relative to gravity have been used as a measure for the precision of verticality perception in the roll plane.[30] As reported by others, the SD’s within subjects were larger with increasing head roll.[31, 32] Especially during small tilts of the head in the roll plane, the perception of earth-verticality relies mainly on otolith input as visual, proprioceptive, and semicircular canal input remains similar or are diminished. Trial-to-trial variability can therefore be primarily explained by the properties of the otolith afferents [33, 34] and by central processing that is not optimally tuned for head roll-angles distinct from upright [35, 36]. This feeds the impression that sensory input may change its accuracy and precision when roll-tilted, facilitating or compromising self-orientation in space. This makes estimation of direction of the gravitational vector more variable. Mittelstaedt [37] formulated a hypothesis on earth-vertical estimate errors that can explain E-effects. Based on anatomical observations made by Rosenhall [38], Mittelstaedt [37] postulated an imbalance in the tilt signal caused by an unequal number of hair cells in the utriculus and sacculus. The pattern of persistent deviations could be the downside of an optimal strategy for dealing with these imperfections in the head tilt signal. By adding a body-fixed constant vector (idiotropic vector) to the otolith signal, perceived vertical was altered toward the body-longitudinal axis. The model of Mittelstaedt and its recent reinterpretation in terms of optimal perception using Bayesian modelling [30, 31, 39, 40] assume that verticality perception is optimized for small head-roll angles and that the brain makes a presumptive assumption that the head is mostly oriented upright reducing roll overcompensation. However, the small but persistent verticality estimate errors related to increasing head-on-body tilts are still unfavourable. Especially as the signal-to-noise ratios of visual and proprioceptive inputs are thought not to show such roll-angle dependent deviation.[39] Therefore sensory weighting is important in balance preservation as tasks and environmental demands are constantly changing and the brain is depending on the most reliable input source.

In healthy subjects, it can be hypothesized that the presence of more afferent information sources will lead to a more robust estimate and interaction between head and body ensuring a better postural control even in challenging tasks and environments (e.g. vision in complete darkness).[2, 20] In healthy subjects, afferent information will rather be altered by task and environmental restraints than by somatic impairments. In most cases, the presence of multiple afferent sensory input sources will provide sufficient information to overcome the signal-to-noise ratios of vestibular inputs caused by head-on-body tilts in the roll plane, even in the most challenging situations. Therefore, head-on-body tilts resulting in small visual (SVV) and postural deviations (SPV) of verticality will not lead to disturbed postural control in healthy adults. Previous research of our research group have reported SVV and SPV measurements in different head-on-body positions in healthy subjects.[18] The normative data showed tilts of -1.31°(4.46°) (SVV; head-on-body right), 1.30°(4.06°) (SVV; head-on-body position left), -0.23°(1.78°) (SPV: head-on-body position right) and 0.43°(1.82°) (SPV; head-on-body position left). In the present stroke population, we observe a tilt of approximately 6.58° for SVVc total and 1.66° for SPVc total, irrespective of somatosensory loss. This means that stroke survivors encounter more difficulties to estimate the earth vertical which will be explained in the next paragraph.

In stroke survivors afferent input sources can be impaired as a result of brain damage both at the input level or the information processing level, which can lead to large deviations in the internal representation of verticality.[41] When afferent information is absent or altered after neurological damage, problems in postural control can occur even in simple balance tasks. Head-on-body tilt will lead to a deviation of the vertical as visuo-vestibular input signals are altered. When this is combined with somatosensory loss of the body, the extent of the E-effect will increase as fewer alternative input sources are available. Therefore, estimation of the earth vertical is more challenging. This further explains the trial-to-trial variability in stroke subjects during head-on-body tilt, as estimate errors induced by altered otolith signals cannot be counteracted by alternative somatosensory signals from the extravestibular input sources.

At last, one may argue that the E-effect is more prone to influencing variables and that its mechanisms are less understood compared to the A-effect. However, in our study we did not have the equipment to investigate the A-effect. Yet, more important the E-effect is more functional in daily practice. Most balance problems and fall incidents occur when patients are sitting, standing and walking. However, many patients with stroke encounter postural deviations such as head tilts and rotations, and trunk asymmetry (e.g. the pusher syndrome) [27, 42]. Nevertheless, in these situations, tilts are rather small (<40°) and therefore the E-effect is most relevant in daily practice for rehabilitation. It can be stated that the A-effect could be a better discriminator in the underlying mechanisms but in daily practice, rehabilitation experts especially have to deal with the consequences of the E-effect. Therefore, knowledge about the influencing factors and the role of sensory information within the E-effect is crucial for balance recovery.

## Clinical implications

Sensory weighting is crucial for postural control and is necessary to pretune the body and adapt to different tasks and environmental demands. However, in stroke survivors afferent information can be absent, impaired or inadequately processed. Especially the integration of afferent information is challenging for most patients as seen in visuospatial neglect and the pusher syndrome [43]. As a result, patients will rather rely on one specific input source and therefore lose the ability to switch between sensory strategies to keep balance. As often seen in rehabilitation, patients will use primarily visual information to provide orientation of the body in space as a compensatory mechanism for the inability to up- or deweight sensory information.

In normal situations, the vestibular system combined with proprioception of the neck provides information about the interaction between the trunk and a freely moving head. This interaction between the head and trunk is important for the postural control, as well as maintaining the horizontal gaze during walking. When input sources are biased after stroke, people become more dependent of visual information. In order to increase the reliability of the visual information, people after stroke will often have a rigid trunk characterized by a decreased dissociation between the head and the shoulder- and pelvic girdle. This will help them stabilise their gaze as they diminish the total degrees of freedom of the body. A normal selective head and trunk movement during walking is more difficult for the patient just because of this degrees-of-freedom problem. Yet, rigidity of head and trunk on its turn will decrease options in balance recovery strategies leading to a higher risk of falling. Therefore, selective movement of head and trunk is crucial for normal walking in humans but is in our opinion impossible to achieve when the input or processing of afferent information is impaired. Therefore, in clinical practice, sensory retraining strategies combined with cognitive rehabilitation focussing on information processing and attention are key as they can have immediate effects on motor behaviour and balance recovery after stroke.

In addition, more research is needed to provide further insights in therapy to enhance sensory reweighting strategies as a part of balance training.

## Conclusion

The E-effect in verticality perception is present in stroke survivors and is negatively influenced by somatosensory loss. When impairments occur as a result of brain damage, especially on the sensory input and processing level, fewer alternatives are available to pretune and adapt the body to postural perturbations. Clinical rehabilitation should also focus on sensory retraining strategies combined with cognitive rehabilitation to increase balance recovery after stroke.
